# Effects of renal denervation on kidney function in patients with chronic kidney disease: a systematic review and meta-analysis

**DOI:** 10.1038/s41371-023-00857-3

**Published:** 2023-09-04

**Authors:** Ahmed A. Mohammad, Khaled Nawar, Olivia Binks, Mohammed H. Abdulla

**Affiliations:** 1 School of Medicine, University College Cork, Cork, Ireland; 2 Department of Physiology, University College Cork, Cork, Ireland

**Keywords:** Hypertension, Chronic kidney disease

## Abstract

The present study aims to evaluate the clinical outcomes following renal denervation (RDN) for hypertensive patients with chronic kidney disease (CKD). Prospective studies published between January 1, 2010 and November 15, 2022 where systematically identified for RDN outcomes on office and ambulatory blood pressure, estimated glomerular filtration rate (eGFR), creatinine and procedural characteristics from three online databases (Medline, PubMed, EMBASE). Random effects model to combine risk ratios and mean differences was used. Where possible, clinical outcomes were pooled and analyzed at 6, 12 and 24 months. Significance was set at *p* ≤ 0.05. 11 prospective trials, with a total of 226 patients with treatment resistant HTN receiving RDN met the inclusion criteria. Age ranged from 42.5 ± 13.8 to 66 ± 9. Main findings of this review included a reduction in systolic and diastolic office blood pressure at 6 [−19.8 (*p* < 0.00001)/−15.2 mm Hg (*p* < 0.00001)] and 12 months [−21.2 (*p* < 0.00001)/−9.86 mm Hg (*p* < 0.0005)] follow-up compared to baseline. This was also seen in systolic and diastolic 24-hour ambulatory blood pressure at 6 [−9.77 (*p* = 0.05)/−3.64 mm Hg (*p* = 0.09)] and 12 months [−13.42 (*p* = 0.0007)/−6.30 mm Hg (*p* = 0.001)] follow-up compared to baseline. The reduction in systolic and diastolic 24-hour ambulatory blood pressure was maintained to 24 months [(−16.30 (*p* = 0.0002)/−6.84 mm Hg (*p* = 0.0010)]. Analysis of kidney function through eGFR demonstrated non-significant results at 6 (+1.60 mL/min/1.73 m^2^, *p* = 0.55), 12 (+5.27 mL/min/1.73 m^2^, *p* = 0.17), and 24 months (+7.19 mL/min/1.73 m^2^, *p* = 0.36) suggesting an interruption in natural CKD progression. Similar results were seen in analysis of serum creatinine at 6 (+0.120 mg/dL, *p* = 0.41), 12 (+0.100 mg/dL, *p* = 0.70), and 24 months (+0.07 mg/dL, *p* = 0.88). Assessment of procedural complications deemed RDN in a CKD cohort to be safe with an overall complication rate of 4.86%. With the current advances in RDN and its utility in multiple chronic diseases beyond hypertension, the current study summarizes critical findings that further substantiate the literature regarding the potential of such an intervention to be incorporated as an effective treatment for resistant hypertension and CKD.

## Introduction

Chronic kidney diseases (CKD) represent a leading health burden to millions worldwide [1]. Uncontrolled hypertension (HTN) is a risk factor for the development and progression of CKD [2, 3]. Chronic HTN was shown to affect the smaller renal vessels creating a vicious cycle that ultimately leads to a decline in kidney function [4]. Conversely, the progressive decline in kidney function in CKD in and of itself can lead to impaired blood pressure control [5]. The underlying mechanism in the development of HTN includes the sympathetic nervous system and renin-angiotensin-aldosterone system (RAAS) with both systems also implicated in CKD disease development, progression, and long-term outcomes [6, 7].

The general principles in the management of CKD includes blood pressure management vital to the prevention of progression to end stage kidney disease (ESKD) and reduce the relatively high cardiovascular risk within this cohort [5, 8]. Current therapeutic strategies for HTN include the utility of pharmacological and lifestyle interventions, however, multiple limitations have been addressed in the literature. The main shortcoming of such regimens is their limited ability to combat resistant HTN that is often seen in this cohort [9]. Additionally, challenges such as patient tolerability to anti-hypertensive medication as well as adherence to treatment prescriptions, have been identified as reasons for the deficiencies in current treatment standards [10]. To this end, the advent or re-introduction of renal denervation (RDN) as a potential strategy to combat treatment resistant HTN in CKD, was mainly due to its ability to dampen the sympathetic nervous system as well as RAAS. The technology involves the utility of endovascular catheter via a percutaneous method to deliver radiofrequency waves directly through the renal artery. Through this approach, a proof-of-concept study was able to demonstrate the efficacy and safety of RDN to denervate/ablate the renal nerves [11, 12].

The effect of RDN on attenuating high blood pressure is hypothesized to be via two main mechanisms. Firstly, through dampening the effect of renal efferent nerves, which thereby increases renal blood flow, and increases urinary sodium and water excretion [13]. Secondly, through the interruption of renal afferents RDN is also implicated in reducing the central sympathetic tone, which consequently contributes to a reduction in total peripheral resistance and hence a decrease in blood pressure [14].

Throughout the last decade, multiple trials have investigated the effect of RDN on HTN and a recent network meta-analysis of 20 randomized controlled trials, including a total of 2152 patients, demonstrated the superiority of RDN in reducing blood pressure compared to sham or antihypertensive therapy alone [15]. To this end, this review aims to evaluate the safety and efficacy of RDN as a potential strategy to aid in the treatment of HTN in the context of CKD and evaluate the effectiveness of the technique on multiple renal function parameters.

## Methods

### Search strategy and inclusion criteria

The Preferred Reporting Items for Systematic Reviews and Meta-Analyses (PRISMA) guidelines and Revised Assessment of Multiple Systematic Reviews guidelines were used to design and conduct the review [16, 17]. Main features of study design included a priori study design; independent analysis and duplication of screening, selection, and data extraction; assessment of study quality and publication bias; and utilizing relevant methods for analysis of study findings [16, 17].

Three online databases (MEDLINE, PubMed, and Embase) were searched for papers published from January 1, 2010, to November 15, 2022. The following keywords were used and were non-specific to allow for an exhaustive search of this novel topic: renal denervation, renal sympathetic denervation, catheter-based renal denervation, kidney denervation, renal artery denervation. Studies that were retrieved from the initial database search were published in English and from human trials. The inclusion criteria were as follows: (1) original research articles, (2) published after January 1, 2010, in English language, (3) level I or level II prospective studies that (4) assessed the effect of RDN on patients with more than three months of an eGFR below 60 ml/min/1.73 m² or signs of kidney damage such as albuminuria persisting for more than three months and (5) diagnosis of HTN. The exclusion criteria were as follows: (1) studies that assessed patients with secondary causes of HTN other than CKD, (2) renovascular anomalies, (3) congestive heart failure, (4) left-ventricular ejection fraction <35%, (5) studies published in non-English language.

### Literature screening

**Fig. 1:**
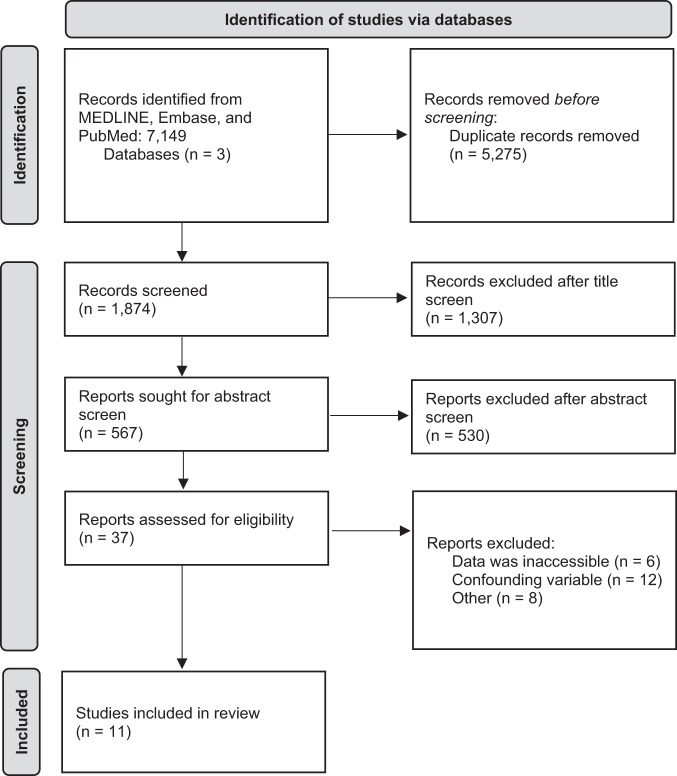
Prisma flow chart of the screening process. A flow chart illustrating the screening procedure for studies to be included or excluded, along with a breakdown of outcomes at each screening phase.

Studies were screened independently and in duplicates by three authors (AAM, KN, OB). Disagreements were internally discussed before moving to the subsequent stage of screening. At all screening stages, including title, abstract and full-text screen, the inclusion, and exclusion criteria was applied, and a PRISMA flow chart was synthesized to summarize the results at each screening stage (Fig. 1) [17]. Agreement between reviewers was assessed at each screening stage to ensure inter-rater reliability via Kappa (k) scores. The k scores were all above the 0.6 threshold which indicates strong inter-rater reliability [18].

### Quality assessment of included studies

Study quality and bias was assessed by two reviewers independently using The Methodological Index for Non-Randomized Studies (MINORS) for non-randomized prospective studies (Supplementary Table 1) [19].

### Data extraction

Data was extracted from the included studies and copied into Excel 2019 (Microsoft, Redmond, WA, USA) to form a homogenous spreadsheet independently and in duplicates. The following fundamental measurements were taken out of the literature if present; author, year of publication, country, trial design, sample size, age, sex of participants, length of follow-up review. Baseline characteristics were also extracted such as office and ambulatory blood pressure, eGFR, and creatinine levels. Periprocedural and post-procedure safety complications, adverse effects, and follow-up data were also extracted. Should a study be comparative the data and outcomes from the control group were not extracted.

### Data analysis

The quantitative results were combined using the statistical program RevMan 5.3 (The Cochrane Collaboration, Copenhagen, Denmark). A random effects study model was used, and inverse variance was employed to weight each study in accordance with the Cochrane Handbook for Systematic Reviews. As a general principle, the pooling of data results required at least three studies. Data heterogeneity must be taken into consideration, and these variations must be reported. *I*^2^ values are utilized to assess heterogeneity caused by variations in study methodologies and populations [20]. Studies with significant levels of heterogeneity (*I*^2^ > 50%) are subjected, as necessary to a sensitivity analysis. This is accomplished by repeating the analysis and substituting the values of the study’s ambiguous and arbitrary experimental decisions [21]. In this meta-analysis, where the random effects model is applied, the degree of variability within the effects is referred to as Tau^2^ and represents the absolute value of true variance. Moreover, given the nature of the meta-analysis and treatment effect, dichotomous variables are compared using relative risk ratios and 95% confidence intervals (CI). Furthermore, standard mean difference (MD) and 95% CI were used to compare continuous variables. In studies where standard deviations were not given, approximative estimate values were reported in accordance with protocol standards [21]. Where applicable, pooled analysis of study outcomes was compared at 6, 12, and 24 months if the outcome was reported across at least two of the three follow-up timepoints.

## Results

### Study characteristics

The PRISMA chart presented in Fig. 1 outlines the results of the literature screen. 11 studies, with a total of 226 patients, were utilized in the synthesis of this analysis [22–32]. Baseline study characteristics are summarized in Table 1. Table 2 outlines the inclusion criteria, CKD stage, surgical characteristics, and methods of drug adherence assessment.

**Table 1:** Study design and baseline characteristics of included Studies.

Author (Year)	Marin (2021) [32]	Scalise (2020) [31]	Ott (2019) [30]	Prasad (2019) [29]	Hameed (2017) [28]	Hering (2017) [27]	Hoye (2017) [22]	Kiuchi (2015) [26]	Ott (2015) [25]	Schlaich (2013) [24]	Hering (2012) [23]
Study Design	Single-arm Prospective	Prospective Comparative	Single-arm Prospective	Single-arm Prospective	Single-arm Prospective	Single-arm Prospective	Single-arm Prospective	Single-arm Prospective	Single-arm Prospective	Single-arm Prospective	Single-arm Prospective
Country	Italy	Italy	Germany	Canada	United Kingdom	Australia	New Zealand	Brazil	Germany	Australia, Europe, USA	Australia
CKD Stage	3,4,5	ESKD	ESKD	3,4	3,4	3,4,5	ESKD	2,3,4	3,4	ESKD	3,4
F/U (months)	12	12	6	24	6	24	12	24	12	12	6
Sample Size	21	12	6	25	11	46	9	30	27	9	15
Age	59.7 ± 17.1	56.5 ± 16.5	42.9 ± 27.9	62.8 ± 12.4	57.4 ± 14.4	66 ± 9	59 ± 9	55 ± 10	63.4 ± 9.4	47.4 ± 13.0^a^	61 ± 9
%Males (*n*)	85.7% (18)	66.7%	67% (4)	NR	72.7% (8)	61% (28)	89% (8)	43% (13)	81% (22)	NR	60% (9)
Number Anti-HTN Drugs	5.50 ± 0.90	4.80 ± 1.20	6.0 ± 0.47	4.9 ± 1.1	3.63 ± 0.84	4.9 ± 1.9	2.63 ± 2.62	4.6 ± 1.4	6.2 ± 1.1	4.20 ± 1.90	5.6 ± 1.3
% Diabetes (*n*)	61.9% (13)	33.3%	17% (1)	NR	45.5% (5)	48% (22)	22% (2)	37% (11)	56% (15)	NR	73% (11)
%CAD (*n*)	NR	NR	NR	NR	NR	28% (13)	NR	17% (5)	37% (10)	NR	NR

Data for age and number anti-HTN drugs are displayed as means and standard deviation (SD).

*CKD* Chronic Kidney Disease, *ESKD* End Stage Kidney Disease, *F/U* Follow-up, *HTN* Hypertension, *NR* Not Reported, *NC* Not Computable, *CAD* Coronary Artery Disease.

^a^Combined mean age for both the RDN and control arm.

**Table 2:** Study methodology and procedural specifications of included studies.

Author (Year)		Marin (2021)^a^ [32]	Scalise (2020)^b^ [31]	Ott (2019) [30]	Prasad (2019) [29]	Hameed (2017) [28]	Hering (2017) [27]	Hoye (2017) [22]	Kiuchi (2015) [26]	Ott (2015) [25]	Schlaich (2013) [24]	Hering (2012) [23]
Inclusion Criteria		1) OSBP > 140 despite being treated with ≥3 anti-HTN medication (including a diuretic)	1) Long-term haemodialysis 2) OBP ≥ 140/90 despite being treated with ≥3 anti-HTN medication (including a diuretic)	1) eGFR<15 2) 4-weeks of stable haemodialysis 3) 24-hour ABP ≥ 135/85, uncontrolled, on 4 weeks of stable treatment (at least three anti-HTN medications)	1) OSBP > 140 despite being treated with ≥3 anti-HTN medication (including a diuretic)	1) eGFR between 15–44 2) OSBP ≥ 140 despite being treated with ≥3 anti-HTN medication	1) eGFR≤60 2) OBP > 140/90 despite being treated with ≥3 anti-HTN medication (including a diuretic if tolerated)	1) Dialysis therapy for at least 3 months 2) OBP > 140/90 despite anti-HTN medication	1) eGFR between 15- 89 (patients with eGFR >60 were required to have microalbuminuria) 2) OSBP ≥ 160 despite being treated with ≥3 anti-HTN medication (including a diuretic)	1) OBP ≥ 140/90 despite being treated with ≥3 anti-HTN medication (including a diuretic)	1) OBP > 140/90, despite being treated with ≥3 anti-HTN medication	NR
CKD Stage	1	0	0	0	0	0	0	0	0	0	0	0
	2	0	0	0	0	0	0	0	19	0	0	0
	3	47.6% (10)	0	0	68.0% (17)	Patients were stage 3 and 4	Patients were stage 3, 4 and 5	0	6	Patients were stage 3 and 4	0	Patients were stage 3 and 4
	4	33.3% (7)	0	0	32.0% (8)	Patients were stage 3 and 4	Patients were stage 3, 4 and 5	0	5	Patients were stage 3 and 4	0	Patients were stage 3 and 4
	5	19.0% (4)	100% (12)	100% (6)	0	0	Patients were stage 3, 4 and 5	100% (9)	0	0	100% (9)	0
% Dialysis		19.0% (4)^c^	100% (12)	100% (6)	0	0	NR	100% (9)	0	0	100% (9)	NR
Dialysis Modality		Haemodialysis	Haemodialysis	Haemodialysis	NA	NA	NR	Haemodialysis (*n* = 6), Peritoneal (*n* = 3)	NA	NA	Haemodialysis	NR
RDN Method		Symplicity Flex (*n* = 4), Symplicity Spyral (*n* = 17)	Bilateral (*n* = 11), Unilateral (*n* = 1, nephrectomy): Symplicity Spyral (*n* = 8), EnligHTN (*n* = 4)	Bilateral: Symplicity Flex	Unilateral (*n* = 4) Bilateral (*n* = 21): Symplicity	Symplicity flex or Symplicity Spyral	Bilateral: Symplicity	Multipolar: EnligHTN	AlCath Flux eXtra Gold Full Circle 2708	Symplicity Flex	Bilateral: Symplicity Flex	Bilateral: Symplicity
Number of Ablations		32.5 ± 15.6	28 ± 4.7	Left: 6.2 ± 4.7 Right: 4.5 ± 0.95	4 to 6 ablations on each side	11.3 ± 2.4	12.9 ± 3.3	Left: 4 Right: 4	Left: 9 ± 3 (5–14) Right: 9 ± 3 (4–14)	≥4	10.2 ± 3.0	9.9 ± 1.5
Drug Adherence Assessment		Direct Questioning	Direct Questioning	NA	NA	NR	Direct Questioning	NR	Review per F/U visit	Urine sampling at 6-month F/U (*n* = 17)	NR	Review per F/U visit

Units for blood pressure and eGFR are in mm Hg and mL/min per 1.73 m^2^ respectively.

*OSBP* Office Systolic Blood Pressure, *HTN* Hypertension, *TRH* Treatment Resistant Hypertension, *eGFR* Estimated Glomerular Filtration Rate, *ABP* Ambulatory Blood Pressure, *CKD* Chronic Kidney Disease, *OBP* Office Blood Pressure, *NR* Not Reported, *NA* Not Applicable, *RDN* Renal Denervation, *F/U* Follow-up.

^a^Only patients with an eGFR < 45 mL/min per 1.73 m^2^ were extracted from this study.

^b^Only RDN treatment arm was extracted from this study.

^c^Only the four patients in stage 5 CKD received dialysis.

Mean ages ranged from 42.5 ± 13.8 to 66 ± 9 years and follow-up ranged from 6 to 24 months. At baseline the number of anti-HTN medications ranged from 2.63 ± 2.62 to 6.2 ± 1.1 (Table 1). Supplementary Table 2 outlines the hypertensive agents by class at baseline within the included studies (Supplementary Table 2). The number of patients in each CKD stage were: 0 stage 1, 19 stage 2, at least 33 stage 3, at least 20 stage 4, at least 40 stage 5. Two studies included patients with stage 3 and 4 CKD but did not report the number in each stage (*n* = 28) and another study included patients with stage 3, 4, and 5 CKD but did not report the number in each stage (*n* = 46) (Table 2).

### Effect of RDN on office blood pressure

#### Systolic office blood pressure at 6 months

**Fig. 2:**
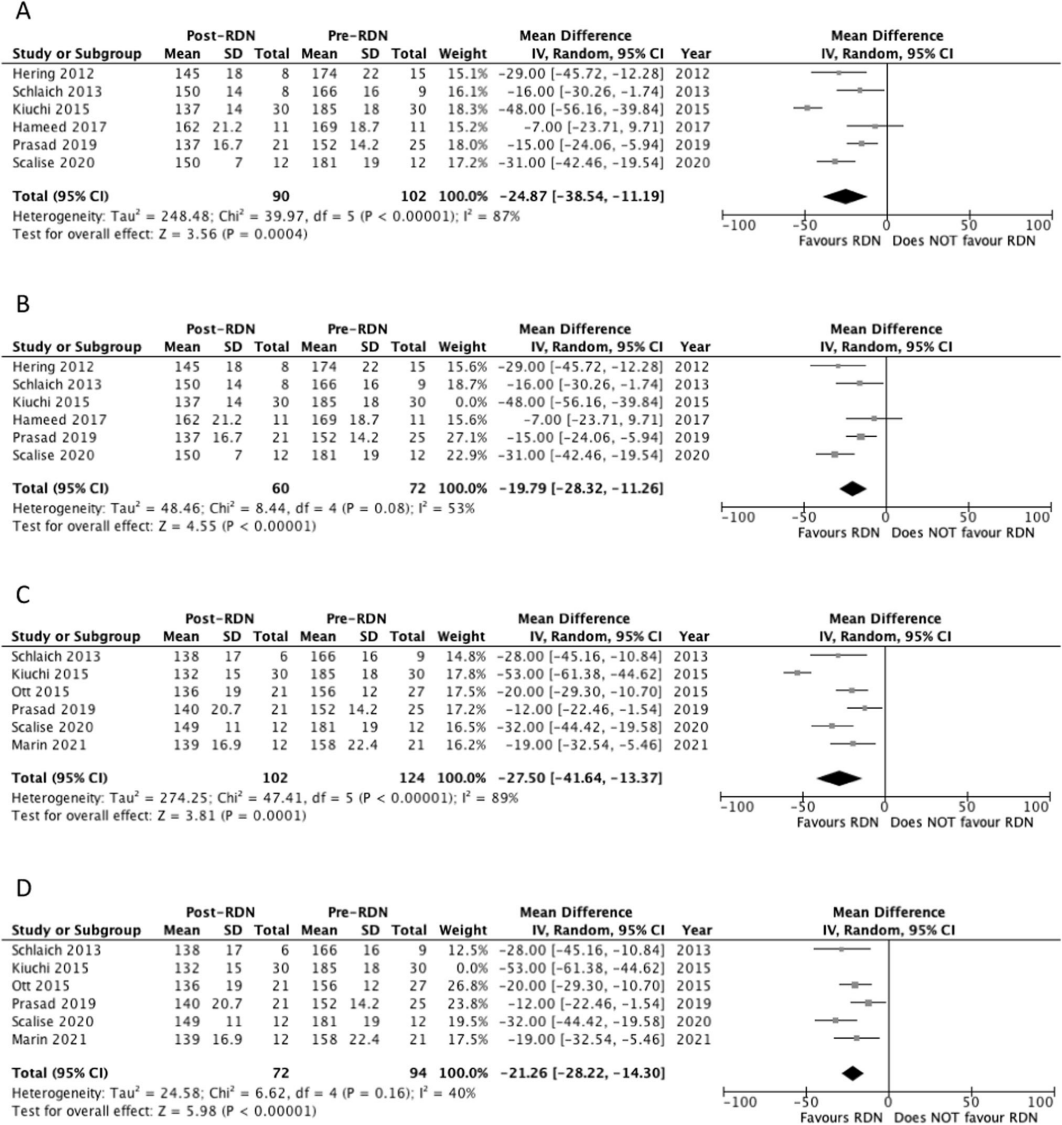
Forest plot of the effects of renal denervation on office systolic blood pressure. **A** 6 month; **B** 6 month sensitivity analysis after removal of Kiuchi 2015; **C** 12 month; **D** 12 month sensitivity analysis after removal of Kiuchi 2015. IV inverse variance, df degrees of freedom.

Six studies reported systolic office blood pressure (OBP) outcomes at 6 months [23, 24, 26, 28, 29, 31]. Five studies reported a significant decrease in OBP at 6 months (Table 3) [23, 24, 26, 29, 31]. Pooled analysis of the studies showed a significant decrease in systolic OBP at 6 months compared to baseline, with MD of −24.9 mm Hg (*p* = 0.0004) and a *I*^2^ value of 87% (Fig. 2A). Heterogeneity was decreased to 53% following removal of Kiuchi 2015 study. Significance was still maintained with the pooled analysis showing a decrease in systolic OBP compared to baseline (MD = −19.8 mm Hg, *p* < 0.00001) (Fig. 2B).

**Table 3:** Office and ambulatory blood pressure outcomes of included studies.

Study Author (Year)	Month (*n*)	Office SBP	*P*-value	Office DBP	*P*-value	Ambulatory SBP	*P*-value	Ambulatory DBP	*P*-value
Marin (2021) [32]	0 (21)	158.0 ± 22.4	-	89.1 ± 15.5	-	157.0 ± 18.0	-	89.8 ± 16.3	-
	12 (12)	−19.42 ± 31.57	NS	−9.50 ± 17.08	NS	−18.0 ± 23.4	NS	−3.87 ± 11.93	NS
Scalise (2020) [31]	0 (12)	181.0 ± 19.0	-	101.0 ± 16.0	-	181.0 ± 20.0	-	100.0 ± 16.0	-
	6 (12)	150.0 ± 7.00	0.0004	82.0 ± 4.00	0.0001	148.0 ± 10.0	0.0004	82.0 ± 4.00	0.0001
	12 (12)	149.0 ± 11.0	0.007	82.0 ± 8.00	0.005	149.0 ± 17.0	0.007	82.0 ± 9.00	0.005
Ott (2019) [30]	0 (6)	-	-	-	-	163.0 ± 16.0	-	96.0 ± 9.00	-
	6 (6)	-	-	-	-	143.0 ± 9.00	0.043	81.0 ± 15.0	0.043
Prasad (2019) [29]	0 (25)	152.0 ± 14.2	-	77.0 ± 14.2		140.2 ± 22.6		65.9 ± 13.4	
	6 (21)	137.0 ± 16.7	<0.001	74.0 ± 14.3	NC	145.0 ± 16.1	NC	66.8 ± 15.4	NC
	12 (21)	140.0 ± 20.7	<0.001	72.3 ± 15.9	NC	143.1 ± 23.6	NC	65.8 ± 18.6	NC
	24 (18)	133.0 ± 14.5	<0.001	74.3 ± 24.1	NC	153.5 ± 22.7	NC	67.9 ± 13.8	NC
Hameed (2017) [28]	0 (11)	169 ± 18.7	-	86.1 ± 17.0	-	155 ± 10.2	-	84.2 ± 17	-
	6 (11)	162 ± 21.2	NS	84.5 ± 17.0	NR	159 ± 16.1	NS	90.8 ± 14.4	NS
Hering (2017) [27]	0 (46)	152.0 ± 27.0	-	77.0 ± 19.0	-	145.0 ± 18.0	-	76.0 ± 11.0	-
	6 (39)	-	-	-	-	142.0 ± 14.0	NS	74.0 ± 11.0	NS
	12 (41)	-	-	-	-	141.0 ± 19.0	NS	73.0 ± 12.0	0.005
	24 (12)	-	-	-	-	134.0 ± 18.0	0.003	71 ± 8.00	0.005
Hoye (2017) [22]	0 (9)	179.0 ± 28.0	-	90.0 ± 17.0	-	173.0 ± 19.0	-	92.0 ± 11.0	-
	12 (5)	-	-	-	-	149.0 ± 17.0	<0.05	90.8 ± 14.4	<0.05
Kiuchi (2015) [26]	0 (30)	185 ± 18.0	-	107 ± 13.0	-	152 ± 17.0	-	93.0 ± 11.0	-
	6 (30)	137.0 ± 14.0	<0.0001	89.0 ± 8.00	<0.0001	134.0 ± 14.0	<0.0001	86.0 ± 11.0	<0.0001
	12 (30)	132.0 ± 15.0	<0.0001	86.0 ± 9.00	<0.0001	133.0 ± 14.0	<0.0001	85.0 ± 10.0	<0.0001
	24 (27)	131.0 ± 15.0	<0.0001	87.0 ± 9.00	<0.0001	132.0 ± 14.0	<0.0001	84.0 ± 12.0	<0.0001
Ott (2015) [25]	0 (27)	156.0 ± 12.0	-	82.0 ± 13.0	-	151.0 ± 12.0	-	80.0 ± 10.0	-
	12 (21)	136.0 ± 19.0	<0.001	74.0 ± 14.0	0.005	143.0 ± 12.0	0.009	76.0 ± 11.0	0.019
Schlaich (2013) [24]	0 (9)	166.0 ± 16.0	-	NC	-	NC	-	NC	-
	6 (8)	150.0 ± 14.0	0.037	NC	-	NC	-	NC	-
	12 (6)	138.0 ± 17.0	0.019	NC	-	NC	-	NC	-
Hering (2012) [23]	0 (15)	174.0 ± 22.0	-	91.0 ± 16.0	-	159.0 ± 14.0	-	85.0 ± 12.0	-
	6 (8)	145.0 ± 18.0	<0.001	77.0 ± 19.0	0.001	154.0 ± 21.0	NS	79.0 ± 11.0	NS

Data for SBP and DBP are displayed as means and standard deviation (SD) in units of mm Hg.

*SBP* Systolic Blood Pressure, *DBP* Diastolic Blood Pressure, *NR* Not Reported, *NS* Not Significant, *NC* Not Computable.

#### Systolic office blood pressure at 12 months

Six studies reported systolic OBP outcomes at 12 months [24, 25, 28, 29, 31, 32]. Four studies reported a significant decrease in OBP at 12 months (Table 3) [24, 26, 29, 31]. Pooled analysis of the studies showed a significant decrease in systolic OBP at 12 months compared to baseline, with MD of −27.5 mm Hg (*p* = 0.0001) and a *I*^2^ value of 89% (Fig. 2C). Heterogeneity decreased to 40% following removal of Kiuchi 2015 study. Significance was still maintained with the pooled analysis showing a decrease in systolic OBP compared to baseline (MD = −21.2 mm Hg, *p* < 0.00001) (Fig. 2D).

#### Diastolic office blood pressure at 6 months

**Fig. 3:**
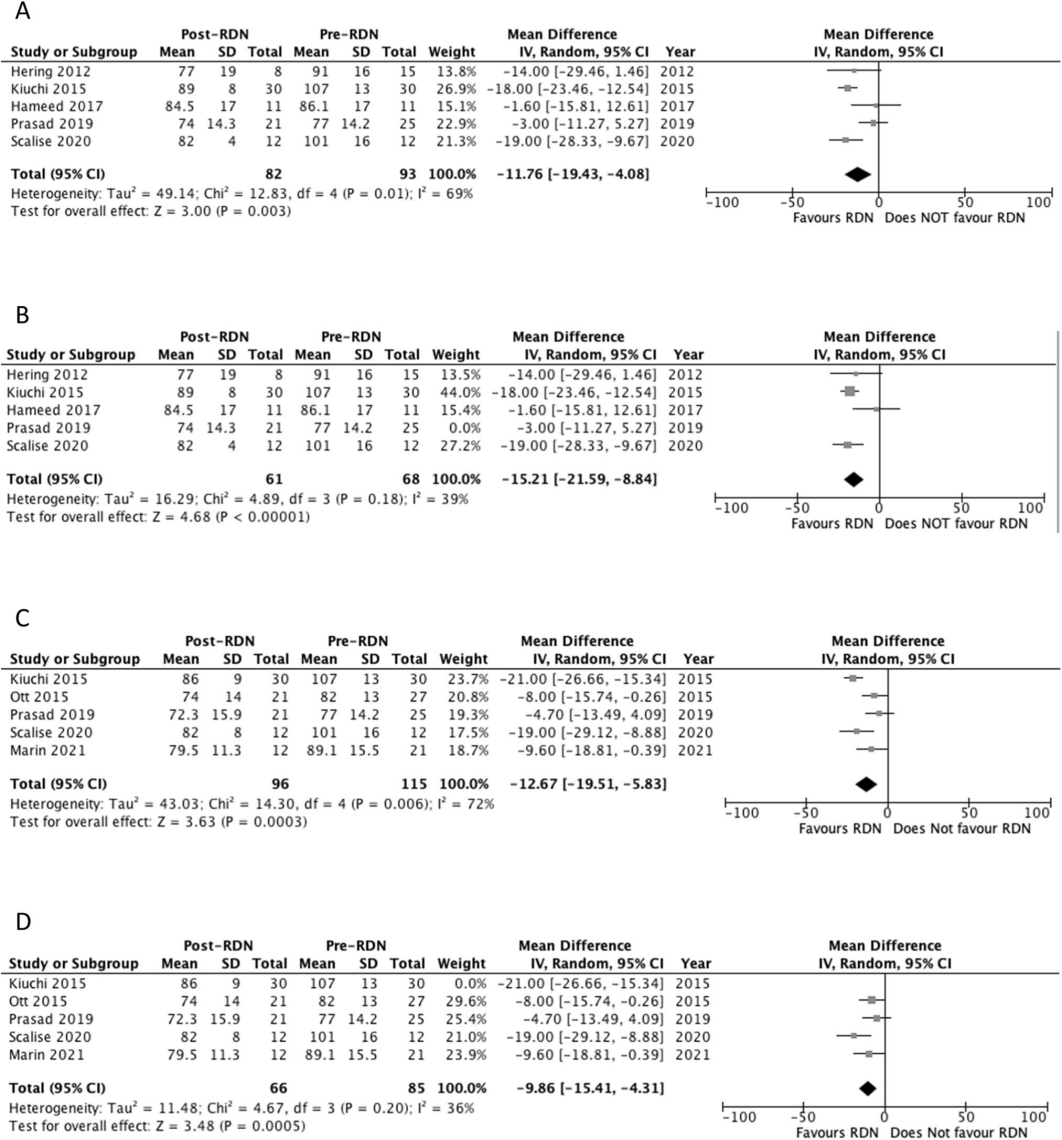
Forest plot of the effects of renal denervation on office diastolic blood pressure. **A** 6 month; **B** 6 month sensitivity analysis after removal of Prasad 2019; **C** 12 month; **D** 12 month sensitivity analysis after removal of Kiuchi 2015. IV inverse variance, df degrees of freedom.

Five studies reported diastolic OBP outcomes at 6 months [23, 26, 28, 29, 31]. Three studies reported a significant decrease in OBP at 6 months (Table 3) [23, 26, 31]. Pooled analysis of the studies showed a significant decrease in diastolic OBP at 6 months compared to baseline, with MD of −11.8 mm Hg (*p* = 0.003) and a *I*^2^ value of 69% (Fig. 3A). Heterogeneity decreased to 39% following removal of Prasad 2019 study. Significance was still maintained with the pooled analysis showing a decrease in diastolic OBP compared to baseline (MD = −15.2 mm Hg, *p* < 0.00001) (Fig. 3B).

#### Diastolic office blood pressure at 12 months

Five studies reported diastolic OBP outcomes at 12 months [25, 26, 29, 31, 32]. Three studies reported a significant decrease in OBP at 12 months (Table 3) [25, 26, 31]. Pooled analysis of the studies showed a significant decrease in systolic OBP at 12 months compared to baseline, with MD of −12.7 mm Hg (*p* = 0.0003) and a *I*^2^ value of 72% (Fig. 3C). Heterogeneity decreased to 36% following removal of Kiuchi 2015 study. Significance was still maintained with the pooled analysis showing a decrease in diastolic OBP compared to baseline (MD = −9.86 mm Hg, *p* = 0.0005) (Fig. 3D).

### Effect of RDN on 24-hour ambulatory blood pressure

#### Systolic 24-hour ambulatory blood pressure at 6 months

**Fig. 4:**
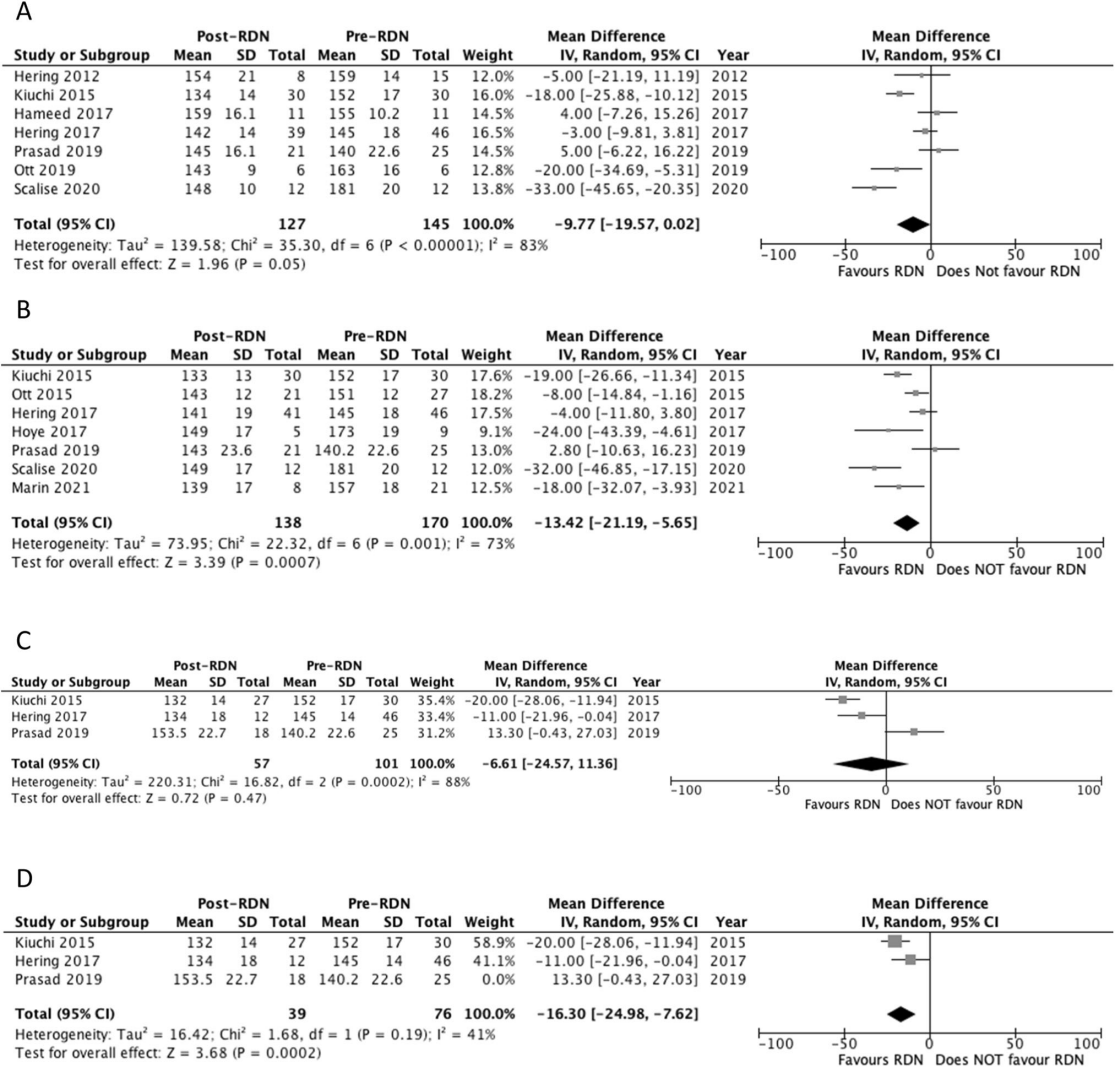
Forest plot of the effects of renal denervation on ambulatory systolic blood pressure. **A** 6 month; **B** 12 month; **C** 24 month; **D** 24 month sensitivity analysis after removal of Prasad 2019. IV inverse variance, df degrees of freedom.

Seven studies reported systolic 24-hour ambulatory blood pressure (ABP) outcomes at 6 months [23, 26–31]. Three studies reported a significant decrease in systolic 24-hour ABP at 6 months (Table 3) [26, 30, 31]. Pooled analysis of the studies showed a significant decrease in systolic 24-hour ABP at 6 months compared to baseline, with MD of −9.77 mm Hg (*p* = 0.05) and a *I*^2^ value of 83% (Fig. 4A). Sensitivity analysis did not reduce heterogeneity or alter significance.

#### Systolic 24-hour ambulatory blood pressure at 12 months

Seven studies reported systolic 24-hour ABP outcomes at 12 months [22, 25–27, 29, 31, 32]. Four studies reported a significant decrease in systolic 24-hour ABP at 12 months (Table 3) [22, 25, 26, 31]. Pooled analysis of the studies showed a significant decrease in systolic 24-hour ABP at 12 months compared to baseline, with MD of −13.42 mm Hg (*p* = 0.0007) and a *I*^2^ value of 73% (Fig. 4B). Sensitivity analysis did not reduce heterogeneity or alter significance.

#### Systolic 24-hour ambulatory blood pressure at 24 months

Three studies reported systolic 24-hour ABP outcomes at 24 months [26, 27, 29]. Two studies reported a significant decrease in 24-hour ABP at 24 months (Table 3) [26, 27]. Pooled analysis of the studies failed to show a significant decrease in 24-hour ABP at 24 months compared to baseline, with MD of −6.61 mm Hg (*p* = 0.47) and a *I*^2^ value of 88% (Fig. 4C). Heterogeneity decreased to 41% following the removal of Prasad 2019 study. The pooled analysis showed a significant decrease in systolic 24-hour ABP compared to baseline, MD of −16.30 mm Hg (*p* = 0.0002) (Fig. 4D).

#### Diastolic 24-hour ambulatory blood pressure at 6 months

**Fig. 5:**
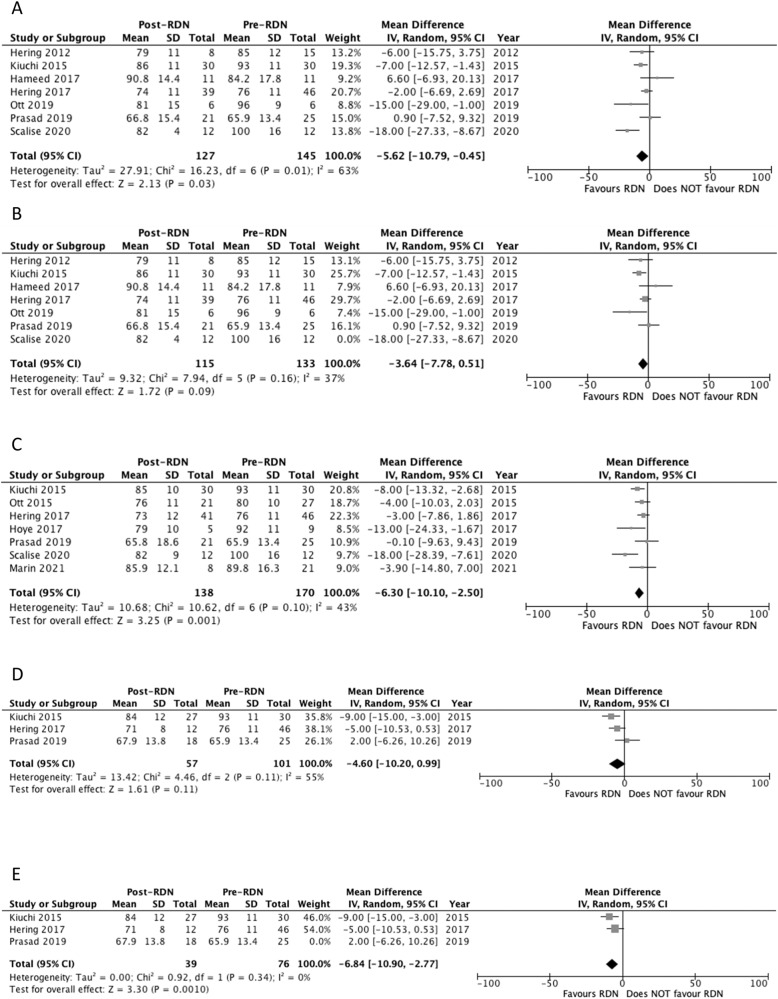
Forest plot of the effects of renal denervation on ambulatory diastolic blood pressure. **A** 6 month; **B** 6 month sensitivity analysis after removal of Scalise 2020; **C** 12 month; **D** 24 month; **E** 24 month sensitivity analysis after removal of Prasad 2019. IV inverse variance, df degrees of freedom.

Seven studies reported diastolic 24-hour ABP outcomes at 6 months [23, 26–31]. Three studies reported a significant decrease in 24-hour ABP at 6 months (Table 3) [26, 30, 31]. Pooled analysis of the studies showed a significant decrease in 24-hour ABP at 6 months compared to baseline, with MD of −5.62 mm Hg (*p* = 0.03) and a *I*^2^ value of 63% (Fig. 5A). Heterogeneity decreased to 37% following the removal of Scalise 2020 study. Significance was no longer maintained following the sensitivity analysis, MD of −3.64 mm Hg (*p* = 0.09) (Fig. 5B).

#### Diastolic 24-hour ambulatory blood pressure at 12 months

Seven studies reported diastolic 24-hour ABP outcomes at 12 months [22, 25–27, 29, 31, 32]. Five studies reported a significant decrease in 24-hour ABP at 12 months (Table 3) [22, 25–27, 31]. Pooled analysis showed a significant decrease in 24-hour ABP at 12 months compared to baseline, with MD of −6.30 mm Hg (*p* = 0.001) and a *I*^2^ value of 43% (Fig. 5C).

#### Diastolic 24-hour ambulatory blood pressure at 24 months

Three studies reported diastolic 24-hour ABP outcomes at 24 months [26, 27, 29]. Two studies reported a significant decrease in 24-hour ABP at 24 months (Table 3) [26, 27]. Pooled analysis of the studies failed to show a significant decrease in 24-hour ABP at 24 months compared to baseline, with MD of −4.60 mm Hg (*p* = 0.11) and a *I*^2^ value of 55% (Fig. 5D). Heterogeneity decreased to 0% following the removal of Prasad 2019 study. The sensitivity analysis showed a significant decrease in diastolic 24-hour ABP compared to baseline, MD of −6.84 mm Hg (*p* = 0.0010) (Fig. 5E).

### Effect of RDN on serum creatinine

#### Creatinine at 6 months

**Fig. 6:**
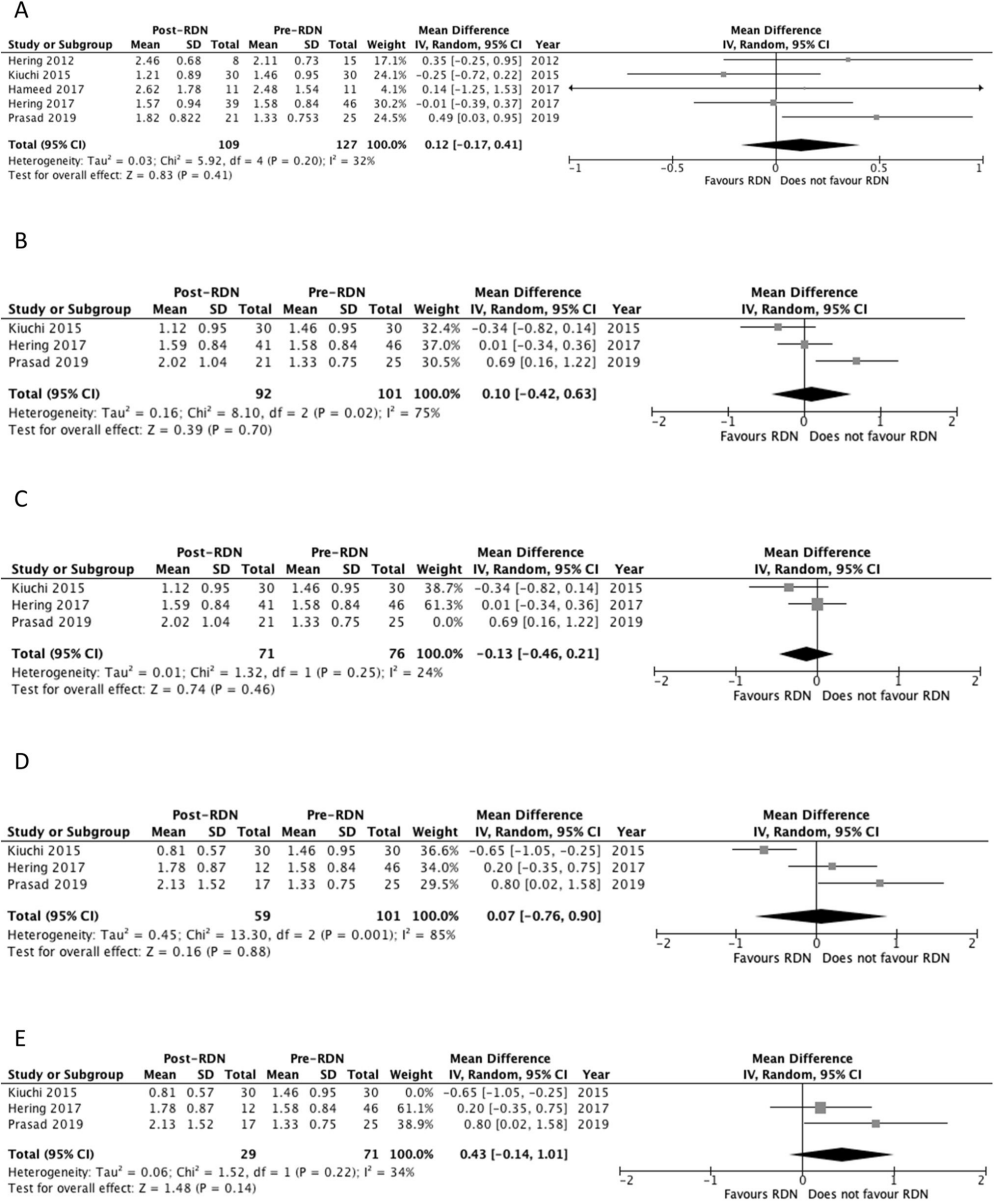
Forest plot of the effects of renal denervation on creatinine. **A** 6 month; **B** 12 month; **C** 12 month sensitivity analysis after removal of Prasad 2019; **D** 24 month; **E** 24 month sensitivity analysis after removal of Kiuchi 2015. IV inverse variance, df degrees of freedom.

Five studies reported creatinine outcomes at 6 months [23, 26–29]. Two other studies reported a significant decrease in creatinine (Table 4) [26, 28]. Pooled analysis of the studies showed no significant difference in creatinine levels at 6 months compared to baseline, with MD of 0.120 mg/dL (*p* = 0.41) and a *I*^2^ value of 32% (Fig. 6A).

**Table 4:** Kidney function outcomes of included studies.

Study Author (Year)	Month (*n*)	eGFR (mL/min per 1.73 m^2^)	*P*-value	Creatinine (mg/dL)	*P*-value	Albumin: Creatinine Ratio (mg/g)	*P*-value
Prasad (2019) [29]	0 (25)	37.0 ± 12.3	-	1.33 ± 0.75	-	530 ± 865	-
	6 (21)	37.2 ± 19.5	NS	1.82 ± 0.82	NS	424 ± 818	NS
	12 (21)	39.9 ± 23.5	NS	2.02 ± 1.04	NS	547 ± 881	NS
	24 (17)	36.0 ± 30.3	NS	2.13 ± 1.52	NS	824 ± 1312	NS
Hameed (2017) [28]	0 (11)	29.4 ± 19.5	-	2.48 ± 1.54	-	2018 ± 2619	-
	6 (11)	25.4 ± 14.4	0.012	2.62 ± 1.78	0.008	1549 ± 2735	NS
Hering (2017) [27]	0 (46)	46.2 ± 13.0	-	1.58 ± 0.84	-	-	-
	6 (39)	48.7 ± 14.6	NS	1.57 ± 0.94.	NS	NR	-
	12 (41)	47.9 ± 14.6	NS	1.59 ± 0.84	NS	NR	-
	24 (12)	46.0 ± 15.2	NS	1.78 ± 0.87	NS	NR	-
Kiuchi (2015) [26]	0 (30)	61.9 ± 23.9	-	1.46 ± 0.95	-	111 ± 120	-
	6 (30)	80.3 ± 35.0	<0.0001	1.21 ± 0.89	<0.0001	45.1 ± 66.6	<0.01
	12 (30)	86.1 ± 35.2	<0.0001	1.12 ± 0.95	<0.0001	44.6 ± 70.5	<0.001
	24 (27)	88.0 ± 39.8	<0.0001	0.81 ± 0.57	<0.0001	14.6 ± 18.8	<0.0001
Ott (2015) [25]	0 (27)	48.5 ± 12.0	-	-	-	-	-
	12 (21)	49.6 ± 15.0	NS	-	-	-	-
Hering (2012) [23]	0 (15)	31.2 ± 8.90	-	2.11 ± 0.73	-	592 ± 955	-
	6 (8)	29.0 ± 7.30	NS	2.46 ± 0.68	NS	355 ± 276	NS

Data for eGFR, Creatinine, and Albumin:Creatinine Ratio are displayed as means and standard deviation (SD).

*eGFR* Estimated Glomerular Filtration Rate, *NS* Not Significant, *NR* Not Reported.

#### Creatinine at 12 months

Three studies reported creatinine outcomes at 12 months [26, 27, 29]. Only one study reported a significant decrease in creatinine (Table 4) [26]. Pooled analysis of the studies showed no significant difference in creatinine levels at 12 months compared to baseline, with MD of 0.100 mg/dL (*p* = 0.70) and a *I*^2^ value of 75% (Fig. 6B). Heterogeneity decreased to 24% following removal of Prasad 2019 study. No change in significance level occurred following the sensitivity analysis (*p* = 0.46) (Fig. 6C).

#### Creatinine at 24 months

Three studies reported Creatinine outcomes at 24 months [26, 27, 29]. Only one study reported a significant decrease in creatinine (Table 4) [26]. Pooled analysis of the studies showed no significant difference in creatinine levels at 24 months compared to baseline, with MD of 0.07 mg/dL (*p* = 0.88) and a *I*^2^ value of 85% (Fig. 6D). Heterogeneity decreased to 34% following removal of Kiuchi 2015 study. No change in significance level occurred following the sensitivity analysis (*p* = 0.14) (Fig. 6E).

### Effect of RDN on eGFR

#### eGFR at 6 months

**Fig. 7:**
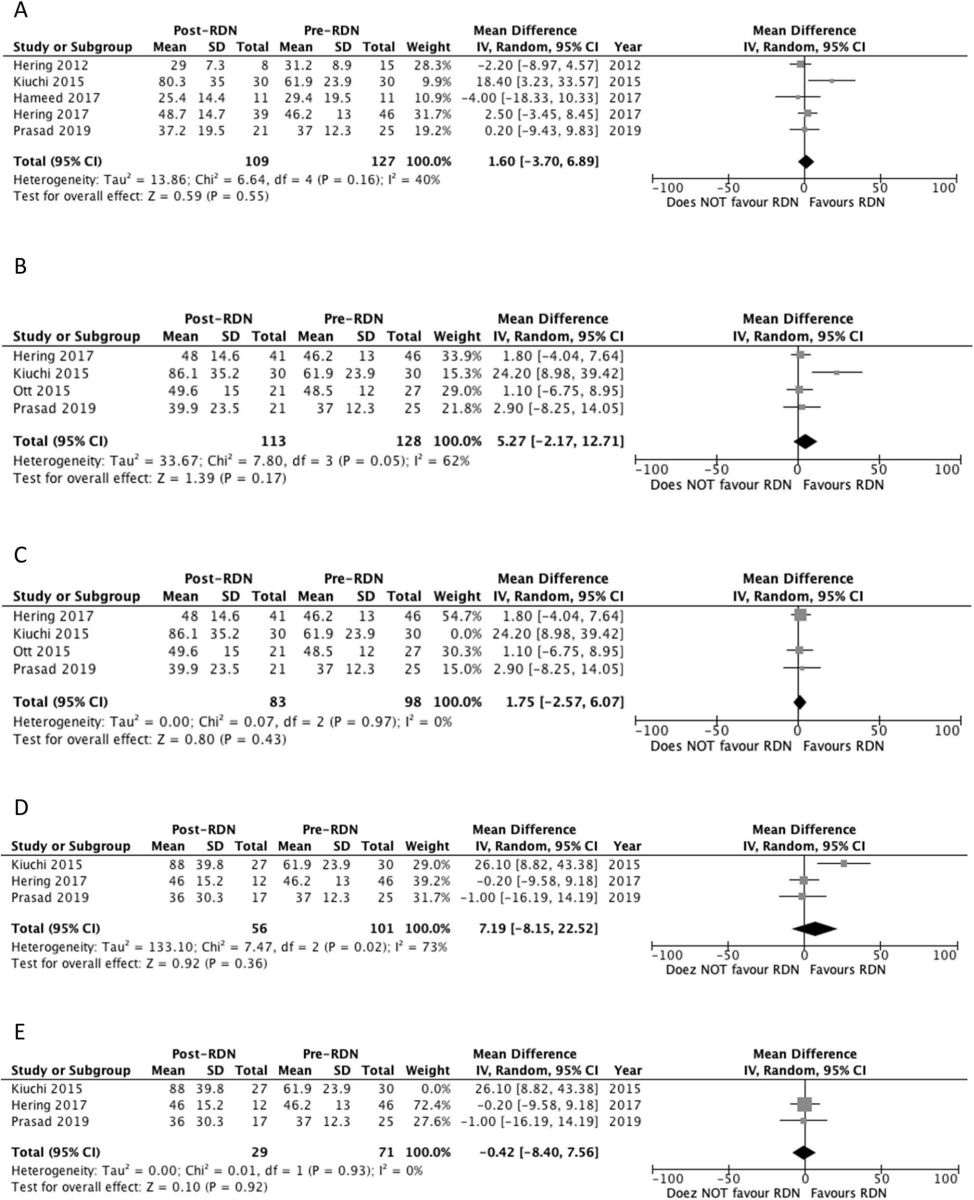
Forest plot of the effects of renal denervation on eGFR. **A** 6 month; **B** 12 month; **C** 12 month sensitivity analysis after removal of Kiuchi 2015; **D** 24 month; **E** 24 month sensitivity analysis after removal of Kiuchi 2015. IV inverse variance, df degrees of freedom.

Five studies reported eGFR outcomes at 6 months [23, 26–29]. Two studies reported a significant increase in eGFR (Table 4) [26, 28]. Pooled analysis of the studies showed no significant difference in eGFR levels at 6 months compared to baseline, with MD of 1.60 mL/min/1.73 m^2^ (*p* = 0.55) and a *I*^2^ value of 40% (Fig. 7A).

#### eGFR at 12 months

Four studies reported eGFR outcomes at 12 months [25–27, 29]. One study reported a significant increase in eGFR (Table 4) [26]. Pooled analysis of the studies showed no significant difference in eGFR levels at 12 months compared to baseline, with a MD of 5.27 mL/min/1.73 m^2^ (*p* = 0.17) and a *I*^2^ value of 62% (Fig. 7B). Heterogeneity decreased to 0% following removal of Kiuchi 2015 study. No change in significance level occurred following the sensitivity analysis (*p* = 0.43) (Fig. 7C).

#### eGFR at 24 months

Three studies reported eGFR outcomes at 24 months [26, 27, 29]. One study reported a significant increase in eGFR (Table 4) [26]. Pooled analysis of the studies showed no significant difference in eGFR levels at 24 months compared to baseline, with MD of 7.19 mL/min/1.73 m^2^ (*p* = 0.36) and a *I*^2^ value of 73% (Fig. 7D). Heterogeneity decreased to 0% following removal of Kiuchi 2015 study. No change in significance level occurred following the sensitivity analysis (*p* = 0.92) (Fig. 7E).

### Effect of dialysis on pooled outcomes

In total 40 patients over five studies received dialysis, with four studies exclusively including patients on dialysis (*n* = 36). All patients received haemodialysis except for three who received peritoneal dialysis (Table 2) [22, 24, 30–32]. No pooled metrics of kidney function included patients receiving dialysis. Pooled analysis of office and 24-hour ambulatory blood pressures that included studies with isolatable dialysis were excluded to assess the impact of dialysis (Table 5). No effect to significance or heterogeneity was demonstrated when sensitivity analysis was performed on office systolic and diastolic blood pressure across 6 and 12 month follow-up (Table 5, Supplementary Fig. 1 and Fig. 2). Sensitivity analysis of 24-hour ambulatory systolic blood pressure however was no longer significant at 6 months with nil effects noted at 12 months (Table 5, Supplementary Fig. 3). Sensitivity analysis of 24-hour ambulatory diastolic blood pressure had nil effect at 6 months but at 12 months demonstrated a reduced heterogeneity while maintaining significance (Table 5, Supplementary Fig. 4).

**Table 5:** Impact of dialysis on pooled outcomes.

Blood pressure	Follow-up	Dialysis Studies (*n*)	Percent of Dialysis Patients in Outcome	Sensitivity Analysis Result
Office Systolic	6 month	Scalise 2020 (*n* = 12), Schlaich 2013 (*n* = 9)	20.6%	Nil effect
	12 month	Marin 2021 (*n* = 4), Scalise 2020 (*n* = 12), Schlaich 2013 (*n* = 9)	20.2%	Nil effect
Office Diastolic	6 month	Scalise 2020 (*n* = 12)	12.9%	Nil effect
	12 month	Marin 2021 (*n* = 4), Scalise 2020 (*n* = 12)	13.9%	Nil effect
24-hour Ambulatory Systolic	6 month	Scalise 2020 (*n* = 12), Ott 2019 (*n* = 6)	12.4%	Significance lost, No change in heterogeneity
	12 month	Marin 2021 (*n* = 4), Scalise 2020 (*n* = 12), Hoye 2017 (*n* = 9)	14.7%	Nil effect
	24 month	NA	NA	NA
24-hour Ambulatory Diastolic	6 month	Scalise 2020 (*n* = 12), Ott 2019 (*n* = 6)	12.4%	Nil effect^a^
	12 month	Marin 2021 (*n* = 4), Scalise 2020 (*n* = 12), Hoye 2017 (*n* = 9)	14.7%	Significance maintained, Heterogeneity reduced
	24 month	NA	NA	NA

Sensitivity analysis results were reported as nil effect if studies with isolatable dialysis were excluded and no change in analysis occurred as compared to the grouped pooled findings.

*NA* Not applicable.

^a^Scalise 2020 was already removed in initial sensitivity analysis of this outcome.

### Complications

Procedural related complications including but not limited to renal artery dissection, hematoma, bleeding, and pseudoaneurysm were extracted (Table 6). No cases of renal artery dissection during the procedure were reported amongst the studies. One patient was noted to experience femoral bleeding post-RDN procedure [26]. Three patients experienced a femoral pseudoaneurysm following RDN procedure, one of which required surgical intervention [22, 24, 32]. Seven patients suffered femoral hematomas [22, 28]. The overall procedural related complication rate was 4.86% (*n* = 226).

**Table 6:** Procedural complications and adverse events of included studies.

Author (Year)	Marin (2021) [32]	Scalise (2020) [31]	Ott (2019) [30]	Prasad (2019) [29]	Hameed (2017) [28]	Hering (2017) [27]	Hoye (2017) [22]	Kiuchi (2015) [26]	Ott (2015) [25]	Schlaich (2013) [24]	Hering (2012) [23]
Renal Artery Dissection During Procedure	0	0	0	0	0	0	0	0	0	0	0
Femoral Hematoma Post-procedure	0	0	0	0	1	0	6	0	0	0	0
Femoral Bleeding Post-procedure	0	0	0	0	0	0	0	1	0	0	0
Femoral Pseudoaneurysm Post-procedure	1	0	0	0	0	0	1	0	0	1	0
Progression to ESRD	0	NA	NA	0	2	0	NA	6	0	NA	0
Dialysis Complications	0	0	0	NA	0	NA	1 (died)	0	0	NR	0
Hospitalization due to BP-related events	0	0	0	0	0	0	0	0	0	NR	0
Myocardial Infarction	0	0	0	0	0	0	1 (4 days post-op)	0	0	NR	0
Death	0	0	0	0	0	0	1	0	0	NR	0

Data reported as number of events.

*ESRD* End Stage Renal Disease, *NA* Not Applicable, *NR* Not Reported, *BP* Blood Pressure.

Additionally, eight patients were observed to progress into ESKD [26, 28]. One patient suffered an unrelated myocardial infarction 4 days post-operatively and another patient died due to dialysis related complications [22].

## Discussion

This review analyzed and pooled the data from 11 studies that performed RDN and met the inclusion criteria outlined consisting of 226 patients with CKD and treatment resistant HTN. Meta-analysis of the data yielded the following results: (1) Stabilization of serum creatinine and eGFR at 6 through 24 month follow-up; (2) A significant reduction in systolic and diastolic 24-hour ABP at 6 through 24 month follow-up; (3) A significant reduction in systolic and diastolic OBP at 6 and 12 month follow-up; (4) Minimal procedural complication related to RDN intervention.

As indicated in both the office and 24-hour ABP results at 12 month follow-up, RDN was seen as a favourable non-pharmacotherapy outcome. The mechanism by which RDN endeavours to lower blood pressure and decrease sympathetic outflow can be attributed to the ablation of the renal sympathetic afferent and efferent nerve fibres [13, 14]. By ablating these signals, excess sympathetic tone is decreased thus helping to conform sympathetic drive. It is understood that the possibility of reinnervation may eventually occur however our ability to significantly assess for this was limited due to few studies that performed follow-up for greater than 12 months [33]. The fundamental principle mechanism by which RDN decreases HTN is based on decreasing activation of the RAAS which then regulates sodium excretion, decreasing the burden of fluid overload. Additionally, vascular resistance is decreased following the direct deactivation of the beta-adrenoceptors on the juxtaglomerular apparatus [34]. Outcomes of a post-hoc analysis of 226 patients from SPYRAL HTN-OFF MED trial emphasized and quantified the decrease of renin and aldosterone levels, supporting the theory of neurogenic crosstalk between renal sympathetic tone and HTN [35].

Previously, treatment resistant HTN patients were considered to be the ideal candidate for RDN therapy. However, evidence from SYMPLICITY HTN-3 and other trials alluded to greater results in patients with moderate or neurogenic HTN. The potential validity of the RDN mechanism is confirmed in other meta-analyses, which looked to determine the efficacy of RDN beyond just HTN in cohorts such as atrial fibrillation, heart failure and obstructive sleep apnoea (OSA) [36–38]. A plethora of poor prognosis conditions are suspected to be associated, as they are directly established due to a hyperactive sympathetic nervous system and presence of neurogenic HTN. As recent evidence suggests in the RADIANCE-HTN SOLO/TRIO and SPYRAL HTN-ON/OFF MED trials, the blood pressure-lowering effect of RDN can potentially be reno- and cardio-protective which is crucial to a CKD patient cohort. Likewise in an atrial fibrillation, heart failure or OSA cohorts previously mentioned [39–42].

As candidate selection criteria for undergoing RDN is still up for consensus within the field, elevated sympathetic nerve activity paired with high blood pressure should be a definitive inclusion [35]. Recognized to be a driver in progression of CKD, this patient cohort may specifically benefit from nephroprotection induced by the sympatholytic effects of RDN responsible for lowering and controlling blood pressure levels. ESKD patients on hemodialysis have been noted to have a significant increase in innervation internally of the renal artery adventitia when compared to other patients with lesser CKD stages or normotensive patients [43]. Recent data from another meta-analysis assessed the correlation between increasing sympathetic tone levels and eGFR and found an inverse relationship across all CKD stages [44].

Current mainstay medical therapies available to interrupt the RAAS pathway for patients with CKD have not shown significant utility in preventing the progressive decline in eGFR [45, 46]. The average decline in eGFR of hypertensive patients was shown to be 2.4 mL/min/1.73 m^2^ per year in a study of 594 patients, in contrast to 1 mL/min/1.73 m^2^ per year in the general population [47, 48]. Additionally, another study demonstrated an increase in decline in eGFR with progressive CKD stages [49]. Thus, while the pooled analysis did not show significance in the increase of eGFR at follow-up, surely RDN was demonstrated to prevent decline and maintain the eGFR in this cohort.

There is also notable promise for nephroprotective and cardioprotective effects from RDN in a CKD cohort. RDN reduces activity of the alpha-adrenoceptors located within the afferent arterioles. Subsequent dilation of these arterioles may result in an improvement in the eGFR [34]. Additionally, renalase secreted by the kidney into the bloodstream, is a protein thought to play a role in optimizing normal cardiac function and blood pressure via catabolism of catecholamines [50]. Renalase activity is significantly decreased in CKD leading to excess catecholamines within the systemic circulation contributing to elevated blood pressure [51]. Thus, RDN may also improve cardiovascular outcomes for these patients by preserving renalase secretion. Moreover, Kiuchi et al. demonstrated significant reduction in the albumin:creatinine ratio (ACR) through to 24 months post-RDN [26]. While the present study was unable to assess the ACR, reductions in albuminuria have been shown to lower risk of progression to ESKD. And thus, RDN may offer additional nephroprotective effects [52].

An essential aspect to consider is the safety of RDN. With direct ablation to the renal vascular in addition to the use of contrast to visualize the vascular the safety of RDN in patients with CKD is especially critical. SYMPLICITY HTN-3 is the largest sham controlled to trial investigate RDN with 535 patients. Throughout the various safety outcomes assessed there were no variance adverse events reported between the intervention and sham group. Notably when the investigators sub-analyzed patients with an eGFR less than 60 mL/min/1.73 m^2^ adverse implications to kidney function was not seen [53]. To this end a recent meta-analysis of 2898 hypertensive patients who received RDN investigated the safety of the procedure on kidney function. The authors reported no significant deleterious effects on kidney function up to 9 months post-RDN [54]. Moreover, when common complications of catheter-based interventions through the femoral artery in addition to unique procedural complications related to RDN were assessed in this review adverse outcomes occurred in 4.86% (*n* = 226) of the patients. Overall, the literature and the findings in this review would suggest favorable safety of RDN for patients with CKD.

### Clinical implications

Indeed, there is a complex pathophysiological mechanism between uncontrolled HTN and CKD defying current medical management. The results of the present meta-analysis as well as the extensive literature presented suggests CKD patients may be the ideal cohort for RDN by providing a reduction of blood pressure and stabilization eGFR and creatinine up to 24 months in patients with treatment resistant HTN. Moreover, there is unique promise for cardiovascular and renal protective effects that warrants further investigation of the utility of RDN in the CKD cohorts. Procedural safety and efficacy have been demonstrated with an overall complication rate of 4.86%.

### Limitations

There are limitations to this meta-analysis that are inherited due to the limited and novel nature of the literature that should be considered when considering the quality of the reported findings. There are no gold standard randomized control studies in the literature that assess the impact of RDN in patients with CKD. Thus, nine of the included studies were prospective observational studies and were prone to bias and have limitations in isolating the impact of RDN [55]. Another notable limitation is the small sample size (*n* = 226) which may lead to over-exaggeration of the impact of RDN on blood pressure and renal function [56].

Moreover, there were numerous factors through which heterogeneity was introduced to the meta-analysis. Including but not limited to procedural methods (number and pattern of ablations, catheter generations, etc) as well as study design, data collection, baseline populations, CKD stages and dialysis, medical therapy, definition of treatment resistant HTN, and the presence or lack of drug adherence assessment. This was seen when pooling of the data was performed and was combated extensively through sensitivity analysis. Sensitivity analysis removed Kiuchi 2015 from 6 pooled outcomes. We anticipate the potential reasons for the heterogeneity pertain to 63% of patients cohort in stage 2 CKD thus having the highest mean eGFR out of all the included studies as well as the use of a novel catheter type. However, it should be noted that in outcomes where sensitivity analysis deemed Kiuchi 2015 to be a source of heterogeneity no change to significance occurred after its removal. Prasad 2017 was removed from 4 pooled outcomes. Sources of heterogeneity introduced by this study likely originate from the cohort having the lowest mean diastolic OBP as well as the lowest mean systolic and diastolic 24-hour ABP. With removal of Prasad 2017 from the pooled analysis of 24-hour ABP at 24 month follow-up significance was achieved for both office and diastolic 24-hour ABP. However, it should be noted that these outcomes only contained two studies post-sensitivity analysis and hence conclusive results pertaining to this outcome should be analyzed with caution. Scalise 2020 included 12 patients in stage 5 CKD receiving dialysis and was identified as a source of heterogeneity in 2 pooled outcomes. Removal from the 6 month diastolic 24-hour ABP analysis caused significance to no longer be achieved with nil effect after its removal from the 12 month diastolic 24-hour ABP analysis.

The results reported throughout this review should be therefore interpreted with caution. Nonetheless, the scarce studies on this novel intervention in a CKD cohort demonstrate homogeneity in terms of the methodology, intervention, and outcomes assessed. Thus, pooled analysis provides a meaningful summary of the literature with increased statistical power and evidence-based data to guide the current stance on RDN and future consensus toward homogenizing larger scale comparative trials.

## Conclusion and future directions

The present study demonstrated that in patients with CKD and HTN the introduction of RDN efficaciously reduces systolic and diastolic OBP up to 12 months, systolic and diastolic 24-hour ABP up to 24 months with statistical significance. Additionally, RDN maintains eGFR and serum creatinine levels at 6, 12, and 24 months follow-up. The results of the pooled analysis suggest an interruption to the progressive decline of kidney function that is typically seen in CKD. Moreover, the safety of RDN in patients with CKD was demonstrated and thus RDN may serve as clinically useful for patients with treatment resistant HTN and CKD. Long-term studies with larger cohorts consisting of randomization and shams that utilize next-generation ablation catheters are required to establish the impact on kidney metrics that expands beyond eGFR and serum creatinine. Future trials should also assess the effect of the blood pressure lowering effects of CKD progression and hence examine whether the effect of RDN on eGFR is dependent on blood pressure reduction or if there is a mechanism independent of blood pressure that contributes to the alterations in eGFR.

### Supplementary information

Supplementary Table and figure

## Data Availability

The original data analysed in this meta-analysis can be found within each respective study included in the pool analysis and is additionally displayed in the present paper.
