# Supplementary material for: Effects of renal denervation on kidney function in patients with chronic kidney disease: a systematic review and meta-analysis
Source: J Hum Hypertens. 2023 Sep 4;38(1):29–44. doi: 10.1038/s41371-023-00857-3 (PMC10803266; doi:10.1038/s41371-023-00857-3)
Supplement: Supplementary file 1 — Supplementary Table and figure [file 41371_2023_857_MOESM1_ESM.pdf]

Supplementary Table 1: MINORS assessment of included non-randomized trials

Study Author (Year)	Martin (2021)	Scalise (2020)	Ott (2019)	Prasad (2019)	Hameed (2017)	Hering (2017)	Hoye (2017)	Kiuchi (2015)	Ott (2015)	Schlaich (2013)	Hering (2012)
A stated aim of the study	2	2	2	2	2	2	2	2	2	2	2
Inclusion of consecutive patients	2	1	1	1	2	1	2	1	1	1	2
Prospective collection of data	2	2	2	2	2	2	2	2	2	1	2
Endpoint appropriate to the study aim	2	2	1	2	2	2	2	2	2	1	2
Unbiased evaluation of endpoints	1	1	1	1	1	1	1	1	1	1	1
Follow-up period appropriate to the major endpoint	2	2	1	2	1	2	2	2	2	2	2
Loss to follow up not exceeding 5%	1	2	2	1	2	1	1	1	1	1	1
Prospective calculation of the study size	1	1	1	1	1	1	1	1	1	1	1
An adequate control group	NA	2	NA	NA	NA	NA	NA	NA	NA	NA	NA
Contemporary groups	NA	2	NA	NA	NA	NA	NA	NA	NA	NA	NA
Baseline equivalence of groups	NA	2	NA	NA	NA	NA	NA	NA	NA	NA	NA
Adequate statistical analyses	NA	2	NA	NA	NA	NA	NA	NA	NA	NA	NA
Total	13/16	21/24	11/16	12/16	13/16	12/16	13/16	12/16	12/16	10/16	13/16

Abbreviations: not applicable (NA)

Each study is scored as follows: 0 (not reported), 1 (reported but inadequate), or 2 (reported and adequate). The maximal score indicating an ideal methodology is 16 if the study is non-comparative studies or 24 if comparative.

Supplementary Table 2: Hypertensive agents by class at baseline of included studies

<table>
<tr><th>Study</th><th>Marin (2021)</th><th>Scalise (2020)</th><th>Ott (2019)</th><th>Prasad (2019)</th><th>Hameed (2017)</th><th>Hering (2017)</th><th>Hoye (2017)</th><th>Kiuchi (2015)</th><th>Ott (2015)</th><th>Schlaich (2013)[a]</th><th>Hering (2012)</th></tr>
<tr><td>Diuretic</td><td>NR</td><td>100% (12)</td><td>100% (6)</td><td>100% (25)</td><td>64% (7)</td><td>89% (41)</td><td>44% (4)</td><td>100% (30)</td><td>85% (23)</td><td>-</td><td>100% (25)</td></tr>
<tr><td>ACEi</td><td>NR</td><td>50% (6)</td><td rowspan="2">100% (6)</td><td>64% (16)</td><td>36% (4)</td><td>46% (21)</td><td>44% (4)</td><td>17% (5)</td><td rowspan="2">96% (26)</td><td>50% (6)</td><td>56% (14/25)</td></tr>
<tr><td>ARB</td><td>NR</td><td>33% (4)</td><td>64% (16)</td><td>45% (5)</td><td>72% (33)</td><td>22% (2)</td><td>83% (25)</td><td>67% (8)</td><td>84% (21)</td></tr>
<tr><td>DRI</td><td>NR</td><td>17% (2)</td><td>-</td><td>-</td><td>-</td><td>-</td><td>-</td><td>10% (3)</td><td>-</td><td>-</td><td>-</td></tr>
<tr><td>MRA</td><td>NR</td><td>58% (7)</td><td>-</td><td>24% (6)</td><td>9% (1)</td><td>37% (17)</td><td>11% (1)</td><td>10% (3)</td><td>11% (3)</td><td>-</td><td>44% (11)</td></tr>
<tr><td>CCB</td><td>NR</td><td>-</td><td>33%(2)</td><td>84% (21)</td><td>100% (11)</td><td>74% (34)</td><td>11% (1)</td><td>93% (28)</td><td>17% (19)</td><td>67% (8)</td><td>92% (23)</td></tr>
<tr><td>Vasodilator</td><td>NR</td><td>42% (5)</td><td>83% (5)</td><td>56% (14)</td><td>-</td><td rowspan="2">63% (29)</td><td>-</td><td>13% (4)</td><td>52% (14)</td><td>50% (6)</td><td>-</td></tr>
<tr><td>AB</td><td>NR</td><td>25% (3)</td><td>83% (5)</td><td>32% (8)</td><td>36% (4)</td><td>11% (1)</td><td>3% (1)</td><td>-</td><td>42% (5)</td><td>20% (5)</td></tr>
<tr><td>AA</td><td>NR</td><td>-</td><td>67%(4)</td><td>40% (10)</td><td>-</td><td>-</td><td>-</td><td>37% (11)</td><td>74% (20)</td><td>42% (5)</td><td>60% (15)</td></tr>
<tr><td>BB</td><td>NR</td><td>83% (10)</td><td>50% (3)</td><td>44% (11)</td><td>36% (4)</td><td>57% (26)</td><td>78% (7)</td><td>80% (24)</td><td>85% (23)</td><td>67% (8)</td><td>40% (10)</td></tr>
</table>

Abbreviations: Not reported; NR, Angiotensin converting enzyme inhibitor; ACEi, Angiotensin receptor blocker; ARB, Direct renin inhibitor; DRI, Mineralocorticoid receptor antagonist; MRA, Calcium channel blocker; CCB, Alpha-receptor blocker; AB , Alpha-receptor agonist; AA, Beta-blocker; BB

All data presented as percent (n)

a - Percentage is of both the renal denervation and control arms of the study

(1A)

Study or Subgroup	Post-RDN Mean	SD	Total	Pre-RDN Mean	SD	Total	Weight	Mean Difference IV, Random, 95% CI	Year	Mean Difference IV, Random, 95% CI
Hering 2012	145	18	8	174	22	15	23.4%	-29.00 [-45.72, -12.28]	2012	
Schlaich 2013	150	14	8	166	16	9	0.0%	-16.00 [-30.26, -1.74]	2013	
Kiuchi 2015	137	14	30	185	18	30	26.7%	-48.00 [-56.16, -39.84]	2015	
Hameed 2017	162	21.2	11	169	18.7	11	23.5%	-7.00 [-23.71, 9.71]	2017	
Prasad 2019	137	16.7	21	152	14.2	25	26.4%	-15.00 [-24.06, -5.94]	2019	
Scalise 2020	150	7	12	181	19	12	0.0%	-31.00 [-42.46, -19.54]	2020	
**Total (95% CI)**			**70**			**81**	**100.0%**	**-25.21 [-45.49, -4.93]**		

Heterogeneity: Tau^2 = 383.81; Chi^2 = 36.53, df = 3 (P < 0.00001); I^2 = 92%
Test for overall effect: Z = 2.44 (P = 0.01)

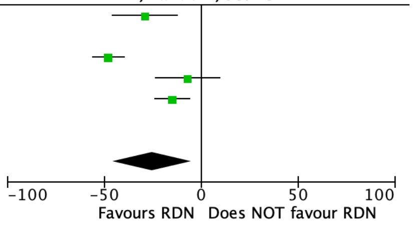

(1B)

Study or Subgroup	Post-RDN Mean	SD	Total	Pre-RDN Mean	SD	Total	Weight	Mean Difference IV, Random, 95% CI	Year	Mean Difference IV, Random, 95% CI
Schlaich 2013	138	17	6	166	16	9	0.0%	-28.00 [-45.16, -10.84]	2013	
Ott 2015	136	19	21	156	12	27	25.4%	-20.00 [-29.30, -10.70]	2015	
Kiuchi 2015	132	15	30	185	18	30	25.6%	-53.00 [-61.38, -44.62]	2015	
Prasad 2019	140	20.7	21	152	14.2	25	25.0%	-12.00 [-22.46, -1.54]	2019	
Scalise 2020	149	11	12	181	19	12	0.0%	-32.00 [-44.42, -19.58]	2020	
Marin 2021	139	16.9	12	158	22.4	21	23.9%	-19.00 [-32.54, -5.46]	2021	
**Total (95% CI)**			**84**			**103**	**100.0%**	**-26.22 [-46.37, -6.07]**		

Heterogeneity: Tau^2 = 393.69; Chi^2 = 47.25, df = 3 (P < 0.00001); I^2 = 94%
Test for overall effect: Z = 2.55 (P = 0.01)

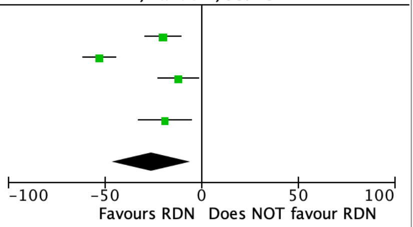

Supplementary Figure 1: Forest plot of the effects of renal denervation on office systolic blood pressure after removal of studies of patients on hemodialysis at (a) 6 month; (b) 12 month. Abbreviations: inverse variance (IV), degrees of freedom (df)

(2A)

Study or Subgroup	Post-RDN Mean	SD	Total	Pre-RDN Mean	SD	Total	Weight	Mean Difference IV, Random, 95% CI	Year	Mean Difference IV, Random, 95% CI
Hering 2012	77	19	8	91	16	15	18.6%	-14.00 [-29.46, 1.46]	2012	
Kiuchi 2015	89	8	30	107	13	30	32.6%	-18.00 [-23.46, -12.54]	2015	
Hameed 2017	84.5	17	11	86.1	17	11	20.2%	-1.60 [-15.81, 12.61]	2017	
Prasad 2019	74	14.3	21	77	14.2	25	28.6%	-3.00 [-11.27, 5.27]	2019	
Scalise 2020	82	4	12	101	16	12	0.0%	-19.00 [-28.33, -9.67]	2020	
**Total (95% CI)**			**70**			**81**	**100.0%**	**-9.66 [-19.19, -0.12]**		

Heterogeneity: Tau^2 = 64.78; Chi^2 = 11.26, df = 3 (P = 0.01); I^2 = 73%
Test for overall effect: Z = 1.99 (P = 0.05)

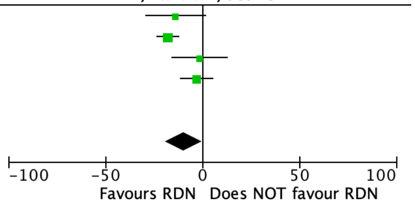

(2B)

Study or Subgroup	Post-RDN Mean	SD	Total	Pre-RDN Mean	SD	Total	Weight	Mean Difference IV, Random, 95% CI	Year	Mean Difference IV, Random, 95% CI
Kiuchi 2015	86	9	30	107	13	30	28.2%	-21.00 [-26.66, -15.34]	2015	
Ott 2015	74	14	21	82	13	27	25.2%	-8.00 [-15.74, -0.26]	2015	
Prasad 2019	72.3	15.9	21	77	14.2	25	23.6%	-4.70 [-13.49, 4.09]	2019	
Scalise 2020	82	8	12	101	16	12	0.0%	-19.00 [-29.12, -8.88]	2020	
Marin 2021	79.5	11.3	12	89.1	15.5	21	23.0%	-9.60 [-18.81, -0.39]	2021	
**Total (95% CI)**			**84**			**103**	**100.0%**	**-11.26 [-19.34, -3.17]**		

Heterogeneity: Tau^2 = 51.95; Chi^2 = 13.21, df = 3 (P = 0.004); I^2 = 77%
Test for overall effect: Z = 2.73 (P = 0.006)

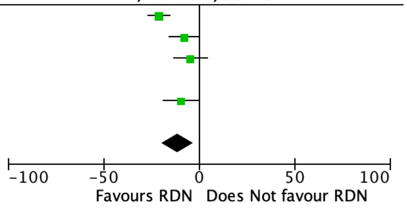

Supplementary Figure 2: Forest plot of the effects of renal denervation on office diastolic blood pressure after removal of studies of patients on hemodialysis at (a) 6 month; (b) 12 months. Abbreviations: inverse variance (IV), degrees of freedom (df).

(3A)

Study or Subgroup	Post-RDN Mean	Post-RDN SD	Post-RDN Total	Pre-RDN Mean	Pre-RDN SD	Pre-RDN Total	Weight	Mean Difference IV, Random, 95% CI	Year
Hering 2012	154	21	8	159	14	15	14.6%	-5.00 [-21.19, 11.19]	2012
Kiuchi 2015	134	14	30	152	17	30	22.8%	-18.00 [-25.88, -10.12]	2015
Hameed 2017	159	16.1	11	155	10.2	11	19.3%	4.00 [-7.26, 15.26]	2017
Hering 2017	142	14	39	145	18	46	23.9%	-3.00 [-9.81, 3.81]	2017
Prasad 2019	145	16.1	21	140	22.6	25	19.3%	5.00 [-6.22, 16.22]	2019
Ott 2019	143	9	6	163	16	6	0.0%	-20.00 [-34.69, -5.31]	2019
Scalise 2020	148	10	12	181	20	12	0.0%	-33.00 [-45.65, -20.35]	2020
**Total (95% CI)**			**109**			**127**	**100.0%**	**-3.82 [-12.80, 5.16]**	

Heterogeneity: $Tau^2 = 75.64$; $Chi^2 = 16.27$, $df = 4$ ($P = 0.003$); $I^2 = 75\%$
Test for overall effect: $Z = 0.83$ ($P = 0.40$)

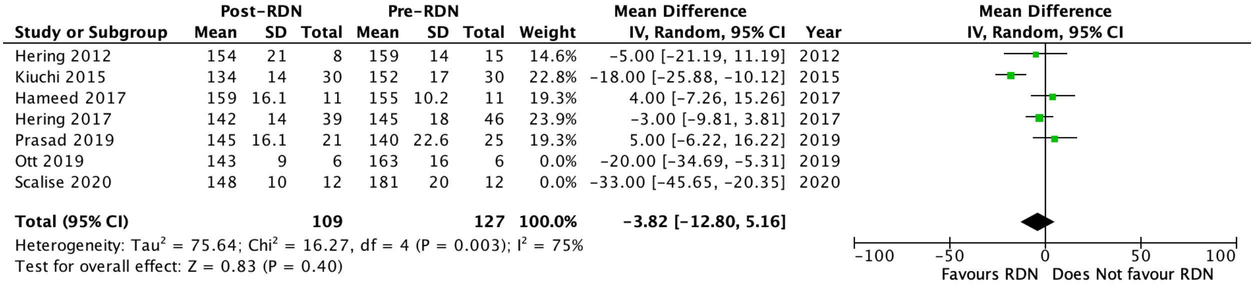

(3B)

Study or Subgroup	Post-RDN Mean	Post-RDN SD	Post-RDN Total	Pre-RDN Mean	Pre-RDN SD	Pre-RDN Total	Weight	Mean Difference IV, Random, 95% CI	Year
Kiuchi 2015	133	13	30	152	17	30	23.1%	-19.00 [-26.66, -11.34]	2015
Ott 2015	143	12	21	151	12	27	24.3%	-8.00 [-14.84, -1.16]	2015
Hering 2017	141	19	41	145	18	46	22.9%	-4.00 [-11.80, 3.80]	2017
Hoye 2017	149	17	5	173	19	9	0.0%	-24.00 [-43.39, -4.61]	2017
Prasad 2019	143	23.6	21	140.2	22.6	25	15.2%	2.80 [-10.63, 16.23]	2019
Scalise 2020	149	17	12	181	20	12	0.0%	-32.00 [-46.85, -17.15]	2020
Marin 2021	139	17	8	157	18	21	14.5%	-18.00 [-32.07, -3.93]	2021
**Total (95% CI)**			**121**			**149**	**100.0%**	**-9.43 [-16.79, -2.07]**	

Heterogeneity: $Tau^2 = 45.75$; $Chi^2 = 12.62$, $df = 4$ ($P = 0.01$); $I^2 = 68\%$
Test for overall effect: $Z = 2.51$ ($P = 0.01$)

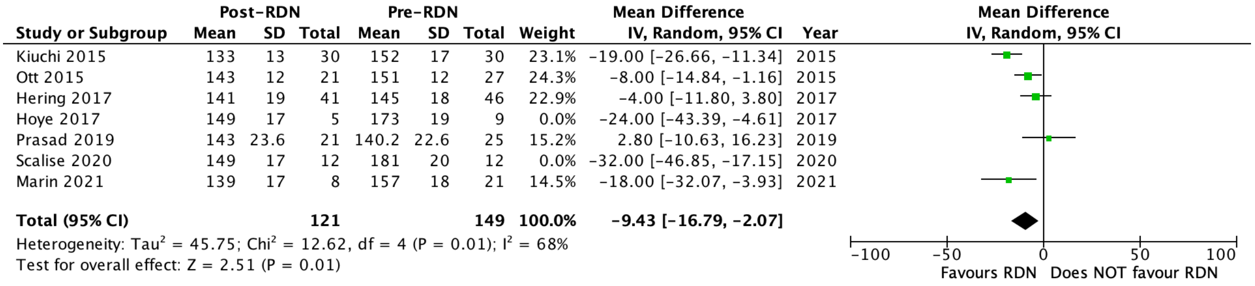

Supplementary Figure 3: Forest plot of the effects of renal denervation on ambulatory systolic blood pressure after removal of studies of patients on hemodialysis at (a) 6 month; (b) 12 months. Abbreviations: inverse variance (IV), degrees of freedom (df).

(4A)

Study or Subgroup	Post-RDN Mean	Post-RDN SD	Post-RDN Total	Pre-RDN Mean	Pre-RDN SD	Pre-RDN Total	Weight	Mean Difference IV, Random, 95% CI	Year
Hering 2012	79	11	8	85	12	15	12.4%	-6.00 [-15.75, 3.75]	2012
Kiuchi 2015	86	11	30	93	11	30	29.1%	-7.00 [-12.57, -1.43]	2015
Hameed 2017	90.8	14.4	11	84.2	17.8	11	7.0%	6.60 [-6.93, 20.13]	2017
Hering 2017	74	11	39	76	11	46	35.8%	-2.00 [-6.69, 2.69]	2017
Ott 2019	81	15	6	96	9	6	0.0%	-15.00 [-29.00, -1.00]	2019
Prasad 2019	66.8	15.4	21	65.9	13.4	25	15.8%	0.90 [-7.52, 9.32]	2019
Scalise 2020	82	4	12	100	16	12	0.0%	-18.00 [-27.33, -8.67]	2020
**Total (95% CI)**			**109**			**127**	**100.0%**	**-2.89 [-6.62, 0.83]**	

Heterogeneity: $Tau^2 = 4.38$; $Chi^2 = 5.28$, df = 4 (P = 0.26); $I^2 = 24\%$
Test for overall effect: Z = 1.52 (P = 0.13)

Mean Difference IV, Random, 95% CI

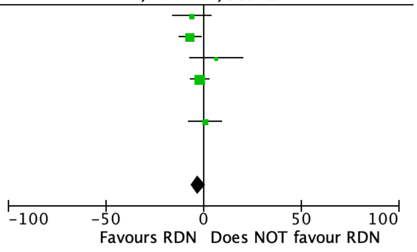

(4B)

Study or Subgroup	Post-RDN Mean	Post-RDN SD	Post-RDN Total	Pre-RDN Mean	Pre-RDN SD	Pre-RDN Total	Weight	Mean Difference IV, Random, 95% CI	Year
Kiuchi 2015	85	10	30	93	11	30	28.4%	-8.00 [-13.32, -2.68]	2015
Ott 2015	76	11	21	80	10	27	22.1%	-4.00 [-10.03, 2.03]	2015
Hering 2017	73	12	41	76	11	46	34.0%	-3.00 [-7.86, 1.86]	2017
Hoye 2017	79	10	5	92	11	9	0.0%	-13.00 [-24.33, -1.67]	2017
Prasad 2019	65.8	18.6	21	65.9	13.4	25	8.8%	-0.10 [-9.63, 9.43]	2019
Scalise 2020	82	9	12	100	16	12	0.0%	-18.00 [-28.39, -7.61]	2020
Marin 2021	85.9	12.1	8	89.8	16.3	21	6.7%	-3.90 [-14.80, 7.00]	2021
**Total (95% CI)**			**121**			**149**	**100.0%**	**-4.44 [-7.28, -1.61]**	

Heterogeneity: $Tau^2 = 0.00$; $Chi^2 = 2.88$, df = 4 (P = 0.58); $I^2 = 0\%$
Test for overall effect: Z = 3.07 (P = 0.002)

Mean Difference IV, Random, 95% CI

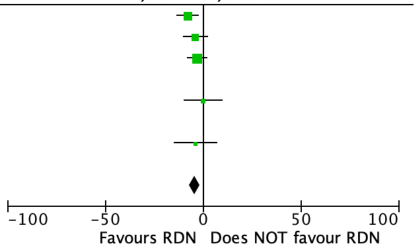

Supplementary Figure 4: Forest plot of the effects of renal denervation on ambulatory diastolic blood pressure after removal of studies of patients on hemodialysis at (a) 6 month; (b) 12 months. Abbreviations: inverse variance (IV), degrees of freedom (df).
